# Tranexamic acid effects in postoperative bleeding outcomes in laparoscopic sleeve gastrectomy: a controlled study

**DOI:** 10.1590/acb370702

**Published:** 2022-10-10

**Authors:** Roger Moura de Brito, Caio Márcio Barros de Oliveira, Ed Carlos Rey Moura, Giuliano Peixoto Campelo, Roclides Castro Lima, Ciro Sousa de Moura Fe, Tércio Maia Sousa, Eduardo José Silva Gomes de Oliveira, Almir Vieira Dibai, Plínio da Cunha Leal

**Affiliations:** 1 Ms. Hospital São Domingos – Center for Bariatric and Metabolic Surgery – Sao Luis (MA), Brazil.; 2 PhD. Universidade Federal do Maranhão – Postgraduate Program in Adult Health – Sao Luis (MA), Brazil.; 3 Graduate student. Universidade Federal do Maranhão – Department of Medicine – Sao Luis (MA), Brazil.

**Keywords:** Bariatric Surgery, Hemorrhage, Tranexamic Acid

## Abstract

**Purpose:**

To demonstrate through a controlled study whether the use of tranexamic acid in bariatric surgeries is effective for bleeding control.

**Methods:**

Prospective, comparative, and double-blind study performed with patients from 18 to 65 years old submitted to bariatric surgery. The selected patients received venous tranexamic acid (TXA) during the induction of anesthesia or not (CG). The anesthesia and thromboprophylaxis protocols were similar among the groups. For statistical analysis, the χ^2^ and analysis of variance tests were performed at a significance level of p < 0.05, using the statistical program SPSS 21.0^®^.

**Results:**

Sixty-one patients were included in the study, 31 in the control group and 30 in the TXA group (GTXA). In the intraoperative period, the bleeding volume was greater in the CG than in the GTXA. In the postoperative period, the tranexamic acid group had a higher value hematocrit, absence of surgical reoperations due to bleeding complications, and shorter hospitalization time than the control group.

**Conclusions:**

The use of tranexamic acid was effective in reducing bleeding rates and of hospital stay length, in addition to demonstrating the clinical safety of its use, for not having been associated with any thromboembolic events.

## Introduction

Obesity is characterized by excessive accumulation of fat in the body, especially in the subcutaneous tissue, compromising health. It is defined as a high-risk chronic disease with serious social and psychological impacts, regardless of age, race, or social class^1^.

Obesity is linked to a multitude of illnesses including type-2 diabetes mellitus, hypertension, heart disease, fatty liver disease, stroke, gallstones, and gastroesophageal reflux disease so presents a major economic burden^2^.

The global prevalence of obesity is alarming, and yet data suggest that these staggering statistics will only continue to rise. Diet, exercise, and medications are often prescribed due to the lower cost and favorable side effect profile, but they produce suboptimal results^3^.

Contrastingly, the field of bariatric surgery has emerged rapidly as an effective treatment strategy in the management of obesity. There is now long-term data supporting the efficacy of weight loss surgery in reducing mortality and morbidity^2^.

Each operation predisposes to specific postoperative complications because of the presence of multiple sequential or crossing staple lines and anastomoses (gastro-entero; entero-entero). The most frequent postoperative complications after bariatric surgery are bleeding, leaks, and stenosis of the anastomosis^4^. Serious short-term complications (leakage and bleeding) occur in 4% of patients^5^.

Laparoscopic sleeve gastrectomy (LSG) is a type of bariatric surgery considered safe and technically simpler to perform than gastric bypass. One of the main risks of this technique is complications in the staple line, which can lead to bleeding and chronic fistulas, among other complications^6^,^7^.

Although advances in methods to reduce complications have already been reported, bleeding in LSG surgeries has still been shown to be a significant problem, affecting mainly patients with previous abdominal surgery^8^,^9^. Bleeding has a higher occurrence in the dissection of the major curvature of the stomach and the stapling line^8^. Several techniques have appeared over time as ways to reduce these complications^10^.

The option of using tranexamic acid (TXA) as an auxiliary in the reduction of bleeding is presented, with the advantage of low cost and fast execution^8^.

TXA is a potent antifibrinolytic that can be used during or after surgery. It prevents the binding of plasminogen to the surface of fibrin and reduces the activation of fibrin, resulting in inhibition of fibrinolysis and minimizing the risk of perioperative bleeding. The dosage of TXA can be determined based on patient’s body weight or renal function, or a standard dose can be given to each patient^11^-^14^.

The meta-analysis study by Ker *et al*.^14^ suggested that a dose of 1 g produced reduction in bleeding that was not improved by giving higher doses and was likely to be sufficient for most adult, having no evidence to support higher doses^13^,^14^.

The use of TXA is already recommended in several surgical contexts, especially for those with an elevated risk of bleeding, and it is recommended by the National Institute of Clinical Excellence (NICE) in surgeries in which the estimated blood loss is greater than 500 mL^15^. Although the volume of bleeding expected in bariatric surgeries, in general, does not reach 500 mL, bleeding is still one of the most feared complications by surgeons^9^.

Even though in the context of bariatric surgeries, the use of TXA is still rare, and there is still uncertainty as to whether TXA may be associated with an increased risk of arterial and venous thrombo-embolism, and this limits its widespread use^8^.

Due to the small number of studies on the use of TXA, it was decided to evaluate its efficacy in the reduction of complications, mainly related to bleeding and thrombotic effects, on the LSG technique.

## Methods

### Study design

This was a prospective, comparative, and double-blind (for the patient and the evaluator) non-randomized clinical trial. This study was approved by the institutional Research Ethics Committee (CAAE 95198518.9.0000.5085/Number 4.058.659) and registered in the Brazilian Registry of Clinical Trials (REBEC RBR-4w39gz).

Patients aged 18 to 65 years old with an American Society of Anesthesiologists (ASA) physical status score of II or III and undergoing bariatric surgery and LSG, from January 2019 to June 2020, in a convenience sampling, were included in the study.

The non-inclusion criteria were as follows: a history of thromboembolic disease or severe comorbidity (ASA IV or more); platelet antiaggregants or anticoagulants; carriers of active intravascular coagulation; acute occlusive vasculopathy; and hypersensitivity to the components of the TXA formula.

Patients underwent general anesthesia with a standardized technique in both groups and monitored with oximetry, non-invasive blood pressure, electrocardiography, and capnography.

The patients included were allocated according to convenience sampling. The selected patients either received venous TXA (1 g) at anesthesia induction (TXA group) or not (control group – CG). The surgeries were performed by the same surgical team. All perioperative care protocols were the same for all groups. Patients received enoxaparin 40 mg for prophylaxis 12 hours before surgery. Both groups received intraoperative fluids (Ringer’s lactate solution) at 2 mL/kg/hour. The surgeons’ team did not routinely place drains postoperatively. Patients were monitored for at least six months to assess the occurrence of possible thromboembolic events.

To help minimize potential bias induced due to non-randomization, the researchers, who collected the data and evaluated patients on the ward, did not know which group the patient belonged to, neither the researcher responsible for the statistical analysis did not know which group each patient belonged to.

### Surgical technique

The surgical technique was the LSG method with six trocars, first the gastroepiploic arcade and greater curvature attachments were divided using a harmonic scalpel. A 32 Fr bougie was used to guide the LSG. The gastric reservoir was created using a linear endoscopic stapler, The Echelon Flex™ Endopath^®^ Stapler (Ethicon Endo Surgery Inc., Cincinnati, OH, United States of America), with cartridges of different heights, depending on the thickness of the stomach. With the entire dissected stomach, the stapler clipped about 2 cm away from the pylorus, usually with a 60-mm green cartridge (4.1 mm). The usual sequence was followed by gold cartridges (3.8 mm) for the antrum and body of the stomach, and then the staple line was finished with blue cartridges (3.5 mm) for the fundus, with the final staple firing 1 cm away from the gastroesophageal junction.

The pneumoperitoneum pressure was maintained between 12-15 mmHg during the entire surgery.

### Discharge criteria

In the study, the Enhanced Recovery After Bariatric Surgery (ERABS) criteria for discharge were used, and all patients were routinely evaluated to check if they met the criteria (Table 1). According to the classification, early discharge = 18 h on the first postoperative day, normal discharge = 8 h on the second postoperative day, and late discharge ≥ 48 h after the end of surgery. All adverse events and complications were recorded^16^.

**Table 1:** Discharge criteria.

**Anamnesis**		
	Pain	VAS score < 4
	Nausea	No complaints of nausea or vomiting
	Intake	> 1 L of fluids within 24 h
	Mobilization	Adequate mobilization
	Calf pain	No complaints of calf pain
	Well-being	Patient feels confident about discharge
**Physical exam**		
	Abdomen	No abdominal tension
	Fever	Body temperature < 38°C
	Heart rate	Frequency < 100 bpm
	Oxygen saturation	SatO2 > 95%
**Laboratory results**		
	Hemoglobin	Post-operative reduction < 2 mmol/L
	Leukocyte count	Leukocyte < 14 × 10^3^/L
	PCR	PCR < 100 mg/L

VAS: visual analogue scale; PCR: polymerase chain reaction.

### Data collection instrument

Social, clinical, and surgical data were collected from medical records. Data from laboratory tests were also collected, such as hemogram, coagulogram, and fibrinogen, at the time that anesthesia was administered and 24 h postoperatively.

Bleeding in the perioperative period was evaluated as intraoperative and postoperative bleeding. Intraoperative bleeding was evaluated by the number of hemostatic interventions performed to control the bleeding points in the staple line. The estimated intraoperative bleeding was assessed by weighing the gauze and the volume of blood found in the suction pump reservoir at the end of the operation (the difference between the total fluid aspirated during surgery minus the amount of fluid infused into the peritoneal cavity during surgery was observed). Postoperative bleeding was evaluated in patients who underwent reoperations or imaging studies in cases of clinical suspicion of bleeding, and a comparative quantitative evaluation was performed of the hematological data obtained by laboratory tests at the time that anesthesia was administered and 24 h postoperatively.

### Statistical analysis

The results were subjected to statistical analysis using SPSS 21.0^®^ software. The following were used for comparisons between groups: χ^2^ test for categorical variables, Student’s t-test for parametrical numerical variables, and Mann-Whitney for non-parametric variables. Normality was verified by the Shapiro-Wilk’s test. Post hoc analysis was performed with the Bonferroni’s test. The level of statistical significance was set at p < 0.05.

## Results

**Figure 1:**
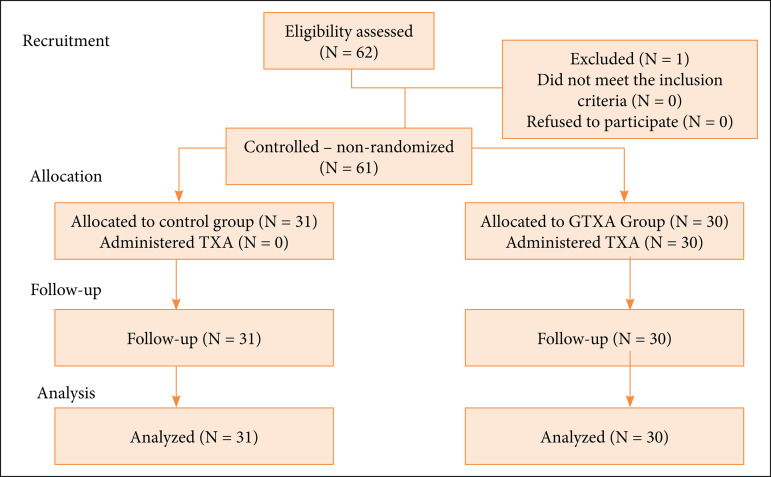
Study consort flowchart.

TXA: tranexamic acid; GTXA: tranexamic acid group.

In total, 61 patients were included in the study, 31 in the control group (CG) and 30 in the TXA group (GTXA), as observed in the flowchart (Fig. 1).

Patients’ clinical characteristics regarding age, body mass index (BMI), gender, comorbidities (diabetes mellitus p = 1.000 and systemic arterial hypertension p = 1.000), use of medications (antihypertensive p = 1.000; hypoglycemic p = 1.000; statin p = 0.255 and PPI p = 1.000), and previous abdominal surgeries were similar among groups. The duration of surgery was similar between the groups (Table 2).

**Table 2:** Clinical characteristics of the patients.

Variables	Groups		
	Control group N = 31	TXA group N = 30	p-value&
Med-age (Min-Max)	39.55 ± 1.71	37.67 ± 2.17	0.497*
Weight (kg)	96.5 ± 1.97	103.9 ± 2.76	0.032*
Height (cm)	160 (154–164)	162.5 (158.8–169.3)	0.045§
BMI	37.3 (36.3–38.7)	38.1 (36.2–40.5)	0.296§
**Sex**			
Female	26 (83.9%)	19 (63.3%)	0.086#
Male	5 (16.1%)	11 (36.7%)	
**Previous surgeries**			
Yes	19 (61.3%)	18 (60%)	1.000#
No	12 (38.7%)	12 (40%)	
Duration of surgery (minutes)	90 (80–100) min	85 (75–95) min	0.224§

* Student’s t-test (mean ± standard deviation);

# Fisher’s test;

§ Mann-Whitney’s test [median (25th percentile - 75th percentile)];

& p < 0.05 was considered for statistical significance; min: minimum; max: maximum; BMI: body mass index; TXA: tranexamic acid.

Hemodynamic variables (heart rate and blood pressure) were similar between groups in all time periods analyzed. In the variables evaluated in the intraoperative period, there was a greater number of staple firings in the GTXA than in the CG (p = 0.013). However, the bleeding volume was greater in the CG than in the GTXA (p = 0.001). There was no difference concerning the weight of the gauzes and number of clips for hemostasis (Table 3).

**Table 3:** Comparison of hemodynamic variables in the pre-, and postoperative periodsand interventions to control intraoperative bleeding between the groups evaluated.

Variables	Groups		p-value&
	Control Group	TXA Group	
Preoperative			
Heart rate (bpm)	73.5 ± 2	75.3 ± 2.19	0.549*
Mean BP (mmHg)	93.3 (76.7–98.7)	83.3 (74.8–93.3)	0.289§
**Intraoperative**			
Heart rate# (bpm)	68.45 ± 1.641	73.27 ± 2.182	0.082§
Average BP# (mmHg)	83.73 ± 2.168	80.61 ± 2.182	0.315§
No. clips for hemostasis	2 (1–6)	5 (2–6)	0.254§
Gauze weight (g)	15 (10–25)	20 (15–25)	0.168§
No. staples	6 (6–6)	6 (6–7)	0.013§
Volume of blood on pump (mL)	80 (80–90)	50 (30–80)	0.001*
**Postoperative**			
Heart rate@ (bpm)	69.45 ± 1.28	72.67 ± 2,01	0.179*
Average BP@ (mmHg)	87 (77–91)	83 (77–91)	0.507§

BP: blood pressure;

* Student’s t-test (mean ± standard deviation);

§ Mann-Whitney’s test [median (25th percentile - 75th percentile)];

# intraoperative;

@ postoperative;

& for statistical significance, p < 0.05 was considered; TXA: tranexamic acid.

When the hematological data such as platelets, PT, AP, activated partial thromboplastin time (TTPa), activated partial thromboplastin time ratio (RTTPa), international normalized ratio (INR) and fibrinogen were analyzed, they were similar between the groups before and after surgery. Although higher values of hemoglobin and hematocrit in the TXAG postoperatively were observed, these data were already higher compared to the CG preoperatively, thus not being possible to perform comparisons of such variables between groups (Table 4).

**Table 4:** Comparison of pre- and postoperative hematological data between the evaluated groups.

Variables	Groups		p-value&
	Control Group	TXA Group	
**Preoperative**			
Hemoglobin (g/dL)	12.3 ± 0.2	13.1 ± 0.3	0.025*
Hematocrit (V/V) %	36.3 (35.2–38.7)	37.2 (36.1–41.2)	0.062§
Platelets	249 (227–280)	243 (218–277)	0.629§
Prothrombin time (s)	14.4 (14.1–14.7)	14.3 (14–4.8)	0.618§
Prothrombin activity (%)	83 (80–87)	86 (79–90)	0.382§
TTPa (s)	31.1 (28.1–32.5)	31.6 (29.7–33.6)	0.310§
RTTPa	1.05 (0.95–1.1)	1.06 (1.01–1.4)	0.352§
INR	1.12 (1.09–1.15)	1.11 (1.06–1.18)	0.493§
Fibrinogen (mg/dL)	277 (274–342)	305 (265–327)	0.234§
**Postoperative**			
Hemoglobin (g/dL)	12.5 (11.8–13)	13.1 (12.2–14.1)	0.037§
Hematocrit (V/V) %	36.48 ± 0.75	38.78 ± 0.65	0.024*
Platelets	266 (232–300)	221 (221–279)	0.387§
Prothrombin time (s)	14.6 (14.3–15)	14.6 (14.1–15)	0.849§
Prothrombin activity (%)	80 (78–84)	82.5 (79–88)	0.071§
TTPa (s)	29.2 (27.9–30.1)	30.5 (29.4–31.9)	0.009§
RTTPa	1.01 (0.95–1.05)	1.03 (1–1.83)	0.053§
INR	1.16 (1.12–1.18)	1.11 (1.07–1.63)	0.052§
Fibrinogen (mg/dL)	309 (288–355)	329 (301–348)	0.283§

TTPa: activated partial thromboplastin time; RTTPa: activated partial thromboplastin time ratio; INR: international normalized ratio;

* Student’s t-test (mean ± standard error);

§ Mann-Whitney [median (25th percentile - 75th percentile)];

& for statistical significance, p < 0.05 was considered; TXA: tranexamic acid.

For a more detailed analysis, we chose to calculate the variation of the hematological data of each patient between the pre- and postoperative periods and then compare between the two groups. There was no difference between the CG and the TXAG when all the hematological variables were compared (Table 5).

**Table 5:** Comparison of the variations of the hematological data of each patientin the pre- and postoperative periods between the groups evaluated.

Variables	Groups		p-value&
	Control group	TXA group	
Hemoglobin variation (g/dL)	0.0032 ± 1.49	-0.1 ± 1.07	0.758*
Hematocrit variation (%)	0.2225 ± 4.22	-0.2916 ± 3.49	0.607*
Platelet variation	-20.000 ± 40.584	-700 ± 38.718	0.062*
Prothrombin activity (%) range	2.96 ± 8.4	2.58 ± 12.7	0.891*
TTPa range (s)	1.36 ± 3.91	0.96 ± 3.33	0.666*
Variation in RTTPa	0.017 ±0.16	0.015 ± 0.11	0.959*
Fibrinogen variation (mg/dL)	-36.45 ± 35.41	-31.63 ± 61.25	0.707*
Prothrombin time (s) range	-0.1 (-0,7–0.2)	-0.3 (-0.6–0.2)	0.707§
INR variation	-0.03 (-0.07–0.02)	-0.015 (-0.05–0.02)	0.382§

TTPa: activated partial thromboplastin time; RTTPa: activated partial thromboplastin time ratio; INR: international normalized ratio;

* Student’s t-test (mean ± standard error);

§ Mann-Whitney [median (25th percentile - 75th percentile)];

& for statistical significance, p < 0.05 was considered; TXA: tranexamic acid.

In the CG, one patient presented a large hematoma of the abdominal wall, which did not require intervention, and another patient required surgical intervention for intra-abdominal bleeding on the first postoperative day.

In the patient who underwent surgical re-approach, there was a drop in hemoglobin from 14.7 to 11.2 g/dL. In addition, attention was drawn to the number of clips used during surgery: 18, in addition to the volume of blood aspirated during surgery: 300 mL. This patient developed hypotension (blood pressure < 90 × 60 mmHg) and heart rate above 120 bpm in the ward, and during the surgical re-approach, on the following day, 300 mL of blood was aspirated.

In the patient with a large wall hematoma, there was a drop in hemoglobin from 12.6 to 9.7 g/dL, and he was treated conservatively, since there was hemodynamic stability.

In both cases, patients were discharged three days after surgery. None of the patients required blood transfusion.

Four patients of the CG had to remain hospitalized for three days. Thus, the time of hospitalization was longer in the CG (median 2; 2-3) than in the GTXA (median 2; 2-2).

No patient in the study presented thrombotic complications related to the use of TXA. Also, no episodes of hematemesis or melena were observed postoperatively.

## Discussion

Bleeding during LSG has already been analyzed from different perspectives by many studies. From an anatomical point of view, the stomach is a richly vascularized organ in which the left and right gastric arteries, gastroepiploic vessels and short gastric arteries supply a large network of submucous plexuses. Thus, bleeding may originate from the division of small branches of the gastroepiploic and short gastric arteries after failure of the power device to seal these vessels. In one study, the main bleeding points were observed in the gastric body^8^.

An increase in bleeding also correlates to longer stapling lines^4^ and stapling lines that require a greater number of fired staples^17^. Additionally, comorbidities, such as liver disease (nonalcoholic fatty liver), coagulopathy, hypertension, and superobesity, increase the risk of bleeding^4^. In a predictive model for hemorrhagic complications of LSG, associations of bleeding with obstructive sleep apnea, the surgeon’s level of experience in bariatric surgery, and the reinforcement of the stapling line, which includes the effectiveness of oversewing the stapling line, were also observed^4^,^18^.

It is difficult to estimate blood loss, especially in procedures with small losses. In this study, intraoperative bleeding from the surgery was quantified by the number of interventions applied to control bleeding (clips or sutures), the volume of blood present in the suction pump, and the weight of the gauze. Confirmation was performed using hematological, preoperative, and postoperative data, as well as other studies related to intraoperative bleeding during LSG^8^,^19^,^20^.

In several surgical contexts, TXA presented positive results, such as in urology, gynecology, cardiology, orthopedics, and traumatology. It reduced bleeding and even the surgical time. Additionally, the safety of the drug was also demonstrated in these clinical trials by the lack of increase in the risk of thromboembolic events^21^-^28^.

Despite the risk of thromboembolic events, the safety of this approach was demonstrated by the absence of thromboembolic events and adverse events in this study, and the patients were monitored for at least six months after surgery. This result was similar to the few studies that used TXA in the context of bariatric surgeries, including the works by Chakravartty *et al*.^8^, who used TXA in the preoperative period of LSG, and by Klaassen *et al*.^12^, who analyzed the use of TXA in patients with postoperative bleeding.

The advantages of using TXA were observed in a clinical study of LSG surgery, mainly with respect to reducing the number of interventions needed to control bleeding, blood loss and surgical time^8^. In this study, the number of interventions applied to control bleeding did not have statistically relevant differences among the groups. The greater number of staples fired in the patients of the TXA group was not a criterion that influenced bleeding or modified the surgical technique, and this was only a circumstantial finding justifiable by the autonomy of the surgeon with respect to the number of staples utilized during the operation; although, it was observed in the literature that a greater number of staples fired may be associated with a greater level of bleeding and complications^17^.

In this study, it was used a single dose of 1 g of TXA, in the same way as Chakravartty *et al*.^8^. Meanwhile, other authors reported higher total doses from 1.5 to 5 g^11^,^12^. It is necessary to state that the results may be affected by an insufficient dosage in obese patients and that studies are needed to define the optimal dosage in obese patients.

Although a significant reduction in the surgical time was observed in another study for patients who used TXA^8^, in this clinical trial, this factor was not statistically relevant.

Although the TXAG presented lower INR and prothrombin time postoperatively, this reduction was not statistically significant. However, in this same group a higher hematocrit value was observed when compared to the CG (p = 0.024), as well as an advantageous reduction in the volume of blood loss, observed by the amount of blood volume in the pump (p = 0.018). A similar result was shown in another study, performed by Chakravartty *et al*.^8^, also in bariatric surgeries.

Well-controlled hemodynamic variables (blood pressure and heart rate) play a fundamental role in the surgical context, including in the identification and management of hemorrhages^12^. Moreover, these parameters are used to evaluate disease control and determine the high-risk factors for bleeding in LSG, such as hypertension^18^. In this study, no significant differences were observed in the mean pressures (such as mean blood pressure) among the groups at any time point. Higher heart rate values were also observed in the TXAG, but this occurred at all the times evaluated, and it is not possible to draw conclusions about this variable in this clinical study.

The length of hospital stay of patients undergoing bariatric surgeries may vary among institutions using different discharge protocols. Although there are already strategies and protocols to reduce the length of hospital stay, such as enhanced recovery after surgery, additional methods for avoiding complications that increase the length of hospital stay, such as bleeding, are continuously being investigated. Considering that bleeding and leakages were the complications mostly commonly reported by studies that investigated the surgical complications of bariatric surgery and had the largest impact on hospital stay, the possibility of improving patient outcomes by intervening in this problem becomes even greater^1^,^20^,^27^,^29^.

In the study performed by Mocanu *et al*.^27^, several strategies were used to avoid bleeding in the stapling line, which proved to be one of the main factors of complications of bariatric surgeries of the sleeve type. TXA and oversewing the stapling line were cited as efficient strategies to prevent complications and shorten the hospital stay.

The group that received intervention (TXA) had shorter hospital stay. This fact was associated with the complications that occurred, including surgical reopening and bleeding of the abdominal wall in two patients in the control group, since they had a length of stay of three days and the other patients only two.

With respect to postoperative complications, including bleeding, which was reflected in the time of hospital stay, the TXAG presented an advantage compared to the CG. It is worth mentioning that bleeding after LSG is associated with increased complications, readmission rates, surgical reoperations, and mortality at 30 days, suggesting the importance of maintaining interventions that reduce the risk of bleeding^8^.

In this case, LSG does not meet the NICE criteria, although the use of TXA may have been assessed as safe and to have positive benefits in this study and in other studies that assessed its use in LSG^8^,^11^,^15^,^28^.

Regarding the strengths of the study, it can be noticed the evaluation of groups submitted to the same surgical procedure and similar anesthetic management. Also, the non-randomization of the groups and the use of convenience sampling were limitations of the study.

## Conclusions

Although the present study did not conclusively demonstrate that the use of TXA reduces the bleeding rate through hematological data, it was observed significant reduction in intraoperative bleeding, with fewer postoperative complications, which led to a reduction in hospital stay. Moreover, it was possible to demonstrate the clinical safety of its use, as it was not associated with any thromboembolic events, which added to the advantages of its low cost and easy applicability, may be an important alternative for the prevention of bleeding in the stapling line of patients undergoing LSG.
